# Electronic Properties of Synthetic Shrimp Pathogens-derived DNA Schottky Diodes

**DOI:** 10.1038/s41598-017-18825-6

**Published:** 2018-01-17

**Authors:** Nastaran Rizan, Chan Yen Yew, Maryam Rajabpour Niknam, Jegenathan Krishnasamy, Subha Bhassu, Goh Zee Hong, Sridevi Devadas, Mohamed Shariff Mohd Din, Hairul Anuar Tajuddin, Rofina Yasmin Othman, Siew Moi Phang, Mitsumasa Iwamoto, Vengadesh Periasamy

**Affiliations:** 1 Low Dimensional Materials Research Centre (LDMRC), Department of Physics, Faculty of Science, University of Malaya, 50603 Kuala Lumpur, Malaysia; 2 Institute of Biological Sciences, Faculty of Science, University of Malaya, 50603 Kuala Lumpur, Malaysia; 3 Department of Chemistry, Faculty of Science, University of Malaya, 50603 Kuala Lumpur, Malaysia; 4 Centre for Research in Biotechnology for Agriculture (CEBAR), University of Malaya, 50603 Kuala Lumpur, Malaysia; 5 High Impact Research (HIR) Functional Molecules Laboratory, Institute of Biological Sciences, Faculty of Science, University of Malaya, 50603 Kuala Lumpur, Malaysia; 6 Institute of Ocean and Earth Sciences, University of Malaya, Kuala Lumpur, 50603 Malaysia; 7 Department of Physical Electronics, Tokyo Institute of Technology, 2-12-1 Okayama, Meguro-ku, Tokyo, 152-8552 Japan; 8 Faculty of Veterinary Medicine, Universiti Putra Malaysia, 43400 Serdang, Selangor Malaysia

## Abstract

The exciting discovery of the semiconducting-like properties of deoxyribonucleic acid (DNA) and its potential applications in molecular genetics and diagnostics in recent times has resulted in a paradigm shift in biophysics research. Recent studies in our laboratory provide a platform towards detecting charge transfer mechanism and understanding the electronic properties of DNA based on the sequence-specific electronic response, which can be applied as an alternative to identify or detect DNA. In this study, we demonstrate a novel method for identification of DNA from different shrimp viruses and bacteria using electronic properties of DNA obtained from both negative and positive bias regions in current-voltage (I–V) profiles. Characteristic electronic properties were calculated and used for quantification and further understanding in the identification process. Aquaculture in shrimp industry is a fast-growing food sector throughout the world. However, shrimp culture in many Asian countries faced a huge economic loss due to disease outbreaks. Scientists have been using specific established methods for detecting shrimp infection, but those methods do have their significant drawbacks due to many inherent factors. As such, we believe that this simple, rapid, sensitive and cost-effective tool can be used for detection and identification of DNA from different shrimp viruses and bacteria.

## Introduction

Since the discovery of the double helix structure of DNA by Watson and Crick in 1953, DNA has been described as the basic building block of life^1,2^. DNA electronics gave birth to the idea, and the possibility of nucleic acid assisted production of the small-scale electronic devices and denser circuits. It has been the subject of intense investigation over the past decade due to its essential role in the operation of electronic devices^3^. In recent years, researchers have utilised new device production methods due to the difficulties and limitations of conventional technologies when it comes to nano-scale processing^4^. Over the past few decades, DNA has been demonstrated to play crucial roles in overcoming these barriers in electronic devices because of its semiconducting-like^5^ or diode-like behavior^6^. This electronic feature of DNA allows possible applications in various fields such as biology, physics, chemistry, computer science and engineering to develop a small, simple, rapid and sensitive electronic device^3^.

Diodes are electronic devices which allow current to flow in one direction only. Common I–V graphs of diodes depict an exponential growth in the current with a small increase in the voltage, also known as rectification behaviour^7^. In this work, we utilise the DNA molecules to create a DNA-metal (semiconductor-metal) device and study the effect of electric current conduction across it. Previous works have indicated that DNA has rectifying properties. By sandwiching the DNA with a metal, we can create a form of diode known as the Schottky diode, which imparts a rectifying effect on an I–V profile as observed with a conventional p-n junction. This DNA-Schottky diode can have potential applications ranging from biosensors to DNA detection and identification devices^6,8–14^.

Aquaculture in shrimp industry has become the fastest food growing sector throughout the world. Countries that are pioneering in shrimp culture are China, Vietnam, Taiwan, India, Thailand, Iran, Ecuador, Philippines, Southeast Asia, Australia and South America^15^. *Penaeus monodon* and *P.varnamei* are the two most dominant species cultivated in Asia Pacific Region. However, shrimp culture in many Asian countries faced a huge economic loss due to disease outbreaks. Nine of these pathogens are viruses responsible for economically crippling shrimp culture industry mainly in Asia but not harmful to human health^16–18^. These viruses include White-spot syndrome virus (WSSV), Hepatopancreatic parvovirus (HPV), Yellowhead-head virus (YHV), Infectious hypodermal and hematopoietic necrosis virus (IHHNV), Mourilyan virus (MOV), Monodon baculovirus (MBV), Gill- associated virus (GAV), Taura syndrome virus (TSV) and Hepatopancreatic parvovirus (HPV)^19^. However, disease outbreaks caused massive mortality and a significant loss to the shrimp cultivation industry (USD 6 billion), mainly from the outbreaks caused by the significant shrimp pathogens including white spot syndrome virus (WSSV)^20^.

The infectious outbreak in shrimp industry is not only attributed to a virus but also bacteria and fungus infection. When researchers are still engaged with the prawn infecting viruses, acute hepatopancreatic necrosis disease (AHPND) outbreaks of shrimp caused by the bacteria, *Vibrio parahaemolyticus* started to surface in China (2009) and spread to Vietnam (2010), Malaysia (2011), Thailand (2012)^21^ and Mexico (2013)^22^. AHPND causes high mortality rate in shrimps characterized by massive sloughing of hepatopancrease epithelial cells in the early stage and bacterial sepsis of the hepatopancrease in the late stage using histopathological examination (NACA, 2012). Some of the Vibrio strains are pathogenic to human including *V. parahaemolyticus* and *V. cholerae* that causes food poisoning^23^. In the meantime, fungus infection caused by *Enterocytozoon hepatopenaei* (EHP) occurred in Thailand in 2004. *Enterocytozoon hepatopenaei* (EHP) is a microsporidian parasite that retards the growth of infected shrimp but does not show any other gross sign of disease that makes the detection harder. EHP produces spores that are found abundant in hepatopancrease, while spore replication occurs in the cytoplasmic tubule epithelial cells^24^.

The detection methods applied for viral, bacterial and fungal diseases in shrimp are roughly similar with minute adjustment. These methods are histopathological examination coupled with light microscopy, polymerase chain reaction (PCR) amplification including nested PCR, real-time PCR and multiplex PCR, fluorescence microscopy, *in situ* hybridisation, monoclonal antibody assay based tests and loop-mediated isothermal techniques (LAMP)^15,25^. However, these methods are not flawless and have some drawbacks in identifying pathogens. Molecular diagnostic techniques have made identification and detection of the virus increasingly accurate. However, these tools are often not readily available for rapid diagnosis in the field or shrimp production facilities. The cost of equipment and technical expertise necessary for these techniques is usually beyond the means of typical commercial shrimp farms. Regarding histopathological examination and clinical signs are the first tests to be carried out to evaluate animal health. These methods are not sensitive for diagnosis because similar symptoms can be caused by many factors. For instance, based on the same general morphology and perinuclear site of accumulation, Boonyaratpalin *et al*.^26^ and Chantanachookin *et al*.^27^ mistakenly reported yellowhead virus (YHV) as baculovirus-like virus using histopathological examination.

Closely related species such as *Vibrio parahaemolyticus* and other *Vibrio* species could induce the pathognomonic histology characteristic of AHPND in histopathology examination^28^. In addition, it is not easy to identify EHP infected shrimp because EHP does not show any gross sign of disease except retarded growth and spores are very small and can only be detected in a highly infected organism using microscopic examination^16^. The major drawback of *in situ *hybridisation  technique is that the method takes time in fixation causing degradation in viral genome especially with RNA extracted from RNA viruses which contributes to the false negative result. Even though PCR technology seems to produce reliable results, the amplified viral genome could produce a false negative result when template concentration or amplicon is lower than the detection limit, and also it requires specific primer design. Advanced PCR technologies such as digital PCR, on the other hand, can produce false positive results as a result of high sensitivity. Monoclonal antibodies based tests such as ELISA, immunoblotting and immunohistochemistry are methods performed by well-certified personnel with special equipment which are not user-friendly to the farmers in aquaculture farms. Rapid dissemination of certain viruses needs to be processed and analysed quickly without any waste of time delivering samples to the respective laboratories.

Multiple viral infections in shrimps are an arising issue. Claydon and his research team found out that wild shrimps *Penaeus monodon* located in Brunei Darussalam waters had numerous viral infections with a combination of IHHNV and MBV^19^. The impact of combined infections of viruses or virus with other pathogens has not yet been assessed and should be further analysed. For instance, WSSV, which causes white spot disease may mask the effect of EHP that retards the growth of the shrimps. New diseases are emerging from time to time where scientists are still investigating the cause of existing pathogens and developing cures. Recently, scientists observed a new pathology symptom where the accumulation of vermiform bodies comprised of aggregated, transformed microvilli (ATM) in the tubule lumens of infected shrimps leading to the disease called white faeces syndrome that affect shrimp growth and survival^29^. The cause of aggregated, transformed microvilli in shrimps is still unknown, but it changes the shrimp growth and survivability.

There are a number of biosensor methods already available for detection of viruses, such as those proposed by Nikolay *et al*.^30^, Anusha Natarajan *et al*.^31^, and Saluma Samanman *et al*.^32^: These methods are electrochemical-based sensors requiring extended preparation steps, complicated processes and metal nanoparticles for functionalization which increase the cost of operation and/or involve chemicals. Our current method, however, consists of acquiring electronic signature signals from semiconducting DNA molecules from various pathogens. The pathogens used in this study are Infectious hypodermal and hematopoietic necrosis virus (IHHNV), Monodon baculovirus (MBV), Taura syndrome virus (TSV), Yellow-head virus (YHV), *Escherichia coli* (*E. coli*) and one of the AHPND-causing strains of *V. parahaemolyticus* (AHPND) for the electronic characterization. The automated method of detection proposed in this work, therefore, offers a simple, rapid, cost-effective and portable solution as compared with other techniques.

The primary step of this method includes the fabrication of a metal-semiconductor (DNA)-metal Schottky barrier diode which has a quantitative electronic profile in response to various DNA sequences when positively or negatively biased. This DNA-specific electronic pattern varies according to base pair differences within different DNA sequences and gives rise to characteristic conductivity and current response to the bias voltage. In this work, we demonstrated a sequence-specific I–V profile in both negative and positive bias voltages and calculated different solid state electronic parameters. These measured parameters constitute the basis for our rapid, sensitive, cost-effective and precise method of detection of various viruses used in this work.

## Results

Table 1 shows the concentration and absorbance ratio at 260 nm to 280 nm of DNA measured using a NanoDrop spectrophotometer. In this experiment, the level of DNA mentioned in Table 1 is the real concentration after preparing DNA sample. Before to the I–V measurement, all DNA samples were diluted to obtain the same concentration level of DNA. The ratio of absorbance at 260 nm to 280 nm was provided to assess the purity of DNA. Pure DNA has an A260/A280 ratio of 1.8 to 2.0, and the A260/A230 ratio should be higher than 1.5, ideally close to 1.8. The lower A260/230 values of the samples indicate contamination of the chaotropic salts, carbohydrates, phenolate ions, and guanidine compounds in the extracted DNA. However, these impurities will not interfere with the subsequent analyses^33,34^.

**Table 1:** The NanoDrop result of the four viruses and two bacterial species investigated in this study. The variations in concentrations did not significantly influence the electronic properties of DNA-Al diodes (supplementary information 2: Figure S3).

Name of Pathogen	Concentration (ng/ul)	A260/A280	A260/A230
MBV	612.9	1.73	1.83
IHHNV	570.2	1.74	1.87
YHV	558.2	1.76	1.86
TSV	518.1	1.74	1.84
AHPND	312.6	1.83	1.03
*E. coli*	630.7	2.15	1.28

**Figure 1:**
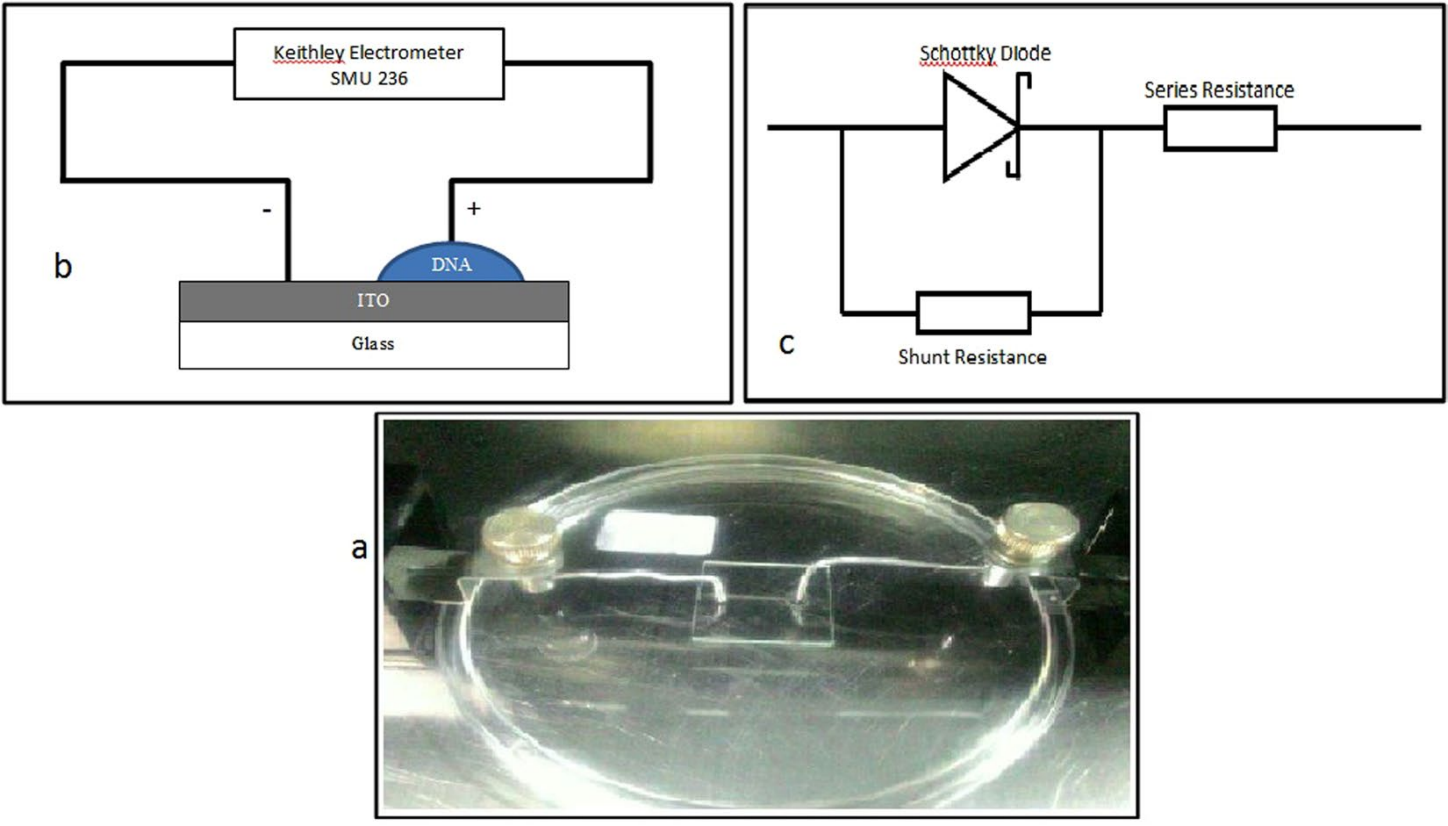
Positive biased I–V profiles for the four types of virus and two bacterial species carried-out in triplicates. A band-like structure is seen for the viruses (top region) and for the bacteria (bottom region).

**Figure 2:**
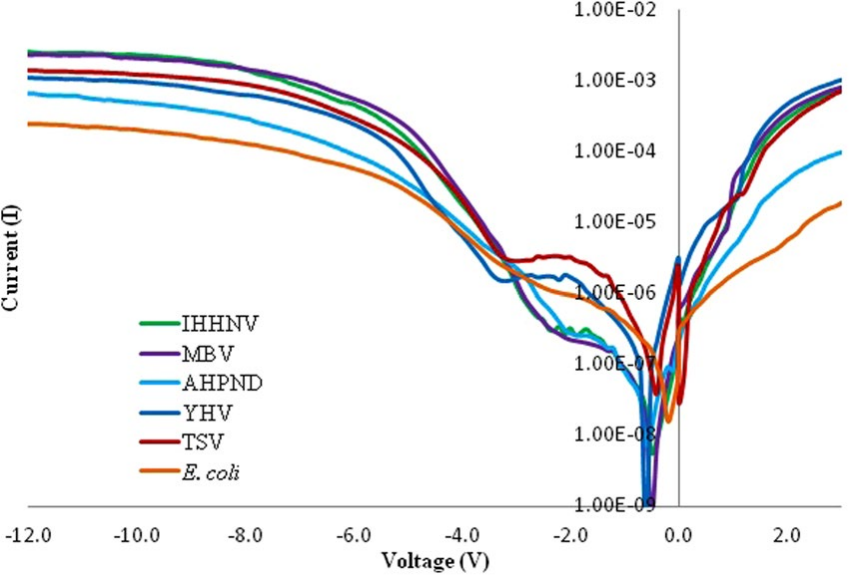
Negative biased I–V profiles for the four types of virus and two bacterial species carried-out in triplicates.

Figures 1 and 2 shows the I–V profile for virus (YHV, TSV, IHHNV and MBV) and bacteria (AHPND and *E. coli*) DNA for forward and reverse bias, respectively. This measurement shows that each profile has significant differences in both positive and negative bias region demonstrating a rectifying effect for all the different types of DNA samples. A band-like structure was observed for the different kinds of organisms tested in this study in the positive region. This pattern demonstrates the potential to utilise the I–V profile as a fingerprint to characterise the difference between virus and bacteria species. The data obtained from IV profile can also be used to calculate other solid-state parameters such as turn-on voltage, series resistance, shunt resistance, ideality factor, barrier height from the positive bias region (Tables 2 and 3) and knee-voltage, breakdown voltage, breakdown current from the negative bias region (Table 4). These values allow for a more in-depth characterisation of each type of DNA based on its base pair sequence electronics.

**Table 2:** Electronic parameters (series resistance (R_s_), barrier height (Ø), ideality factor (n)) calculated for all samples. The voltage range used to calculate values below varies between 1V to 3V due to changes in linear region of the profile obtained in forward bias region.

Samples		*IHHNV*	*MBV*	*AHPND*	*YHV*	*TSV*	*Ecoli*
Method 1	n	13.7	16.8	17.1	12.0	14.6	36.6
	Ø (eV)	0.9091	0.8787	0.9336	0.9140	0.9083	0.9266
	R_s_ (kΩ)	—	—	—	—	—	—
Method 2	n	8.4	6.6	9.7	4.8	9.1	17.9
	Ø (eV)	1.2428	0.8248	0.7158	1.0264	0.9305	0.7764
	R_s_ (kΩ)	1.6	1.9	13.7	1.4	1.9	205

**Table 3:** Electronic parameters (turn-on voltage (V), series resistance (R_s_), shunt resistance (R_p_)) calculated for all samples.

Electronic Parameters	*IHHNV*	*MBV*	*AHPND*	*YHV*	*TSV*	*E. coli*
Shunt Resistance, R_p_ (Ω)	272727.27	186915.89	441803.44	59706.64	1413500.82	568002.52
Series Resistance, R_s_ (Ω)	3952.57	3645.20	31015.77	2903.69	4247.51	156598.00
Turn-on Voltage, V (V)	1.40	0.90	0.85	1.25	1.30	0.60

**Table 4:** Values of knee voltage, breakdown voltage and breakdown current for the negative region.

Electronic Parameters	***IHHNV***	***MBV***	***AHPND***	***YHV***	***TSV***	***Ecoli***
Knee Voltage (V)	−4.80	−4.30	−4.60	−4.70	−4.90	−4.20
Breakdown Voltage (V)	−10.10	−11.80	Not within the voltage range investigated			
Breakdown Current x 10^−3^ (A)	−2.34	−2.08	Not within the voltage range investigated			

**Figure 3:**
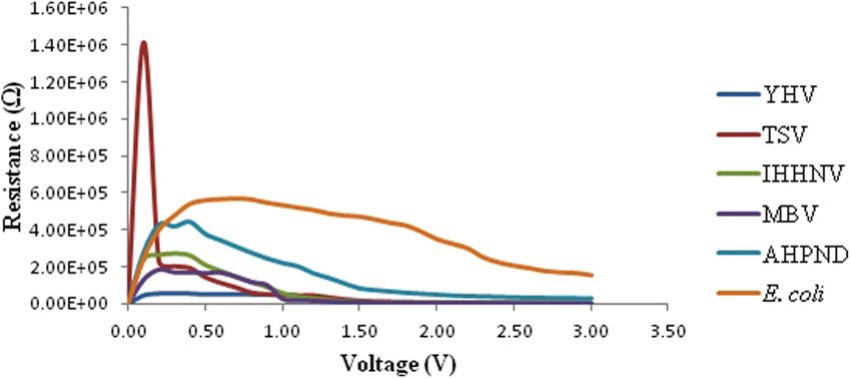
Resistance profile against bias voltage for the four the virus and two bacteria species.

Turn-on voltage observed in Fig. 1 is defined as a certain amount of positive voltage to be applied across diode to operate and conduct current. Each different sample shows its different turn-on voltage. The value of Shunt resistance and series resistances calculated from the I–V profiles (R_S_ = $\partial {\rm{V}}/\partial {\rm{I}}$) are as shown in Tables 2 and 3. Shunt resistance is typically the maximum peak strength obtained from graph while series resistance is lowest resistance value parallel to the x-axis and represents the performance of Schottky-based devices (Fig. 3). In the study, we observed similar trend despite the different series resistance values between the two methods employed. Therefore, identification of various species of virus and bacteria could be achieved based on these parameters which can be utilised as an accurate and efficient method. It will provide an electronic database or profiles for each species which may apply to understand the essentials information for in-depth studies of any DNA species in future.

**Figure 4:**
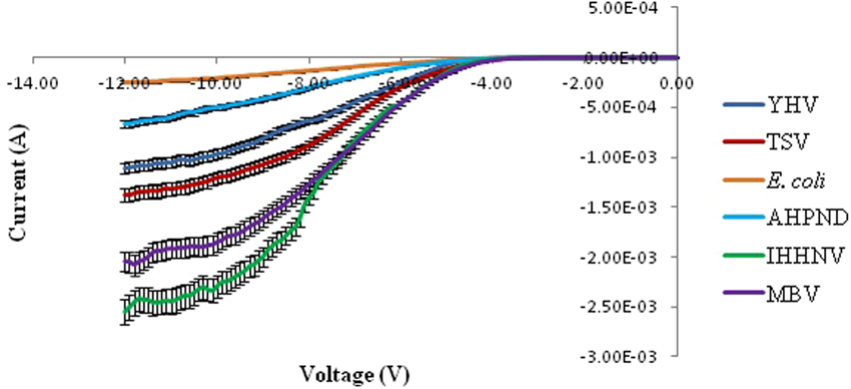
Semi logarithmic I–V characteristics of virus and bacterial species.

Figure 4 shows the experimental I–V characteristics of IHHNV, MBV, AHPND, YHV, TSV and *E. coli* Schottky diode. The low forward bias of I–V measure was used to extract the diode parameter since the current is mostly affected by series resistance and deviates from linearity. The ideality factor, barrier height and series resistance of IHHNV, MBV, AHPND, YHV, TSV and *E. coli* are shown in Tables 2 and 3. Both methods show higher values for the ideality factor, indicating the possible presence of interfacial thin film, barrier inhomogeneity and other phenomena^35,36^. These could lead to some level of uncertainty in IV characteristics measured which shows deviation from ideal Schottky diode in which the values are more significant than 1. The ideality factor determined using Eq. (4) and Eq. (6) in method 1, was relatively larger compared to the values obtained in method 2 using Cheung’s approach with Eq. (7). This variation could be due to the effects of series resistance which was ignored in method 1 which decreases the ideality factor significantly when the effects of series resistance were considered using method 2. As for the barrier height calculated with method 1 using Eqs (4) and (5) shows similar results with method 2 calculated using Eqs (8) and (9), which shows that it was not affected much by the series resistance. Furthermore, the values for series resistance obtained from Cheung’s method using *dV/dlnI* versus *I* and *H(I)* versus *I* plots were in good argument with the values obtained from resistance profile against bias voltage plot. Therefore we suggest Cheung’s method to be the most reliable method to extract diode parameters in this case.

Closer similarities among values of the barrier height will explain how close the species relate to each other, which vary from 0.67 to 0.90, which corresponds between the values generated by Al/p-Si and Al/n-Silicon (Si) Schottky junctions^37^. These values may correspond to the choice of metal contact used and in this work; Al was chosen because of its easy accessibility and cheaper option. However, the significant difference observed in these values may be utilised as one of the characteristic property to identify different DNA sequence. Therefore, each parameter corresponds to specific features related to the charge transfer mechanism within a base sequence, which may provide an electronic database or profiles for each species that may be relevant to a better understanding of the fundamentals about in-depth studies of any DNA species.

## Discussion

Shrimp industry is one of the major food industries in Southeast Asia. Infections caused by virus, bacteria and even fungus are among the crucial risk factors in this industry mainly because they can lead to a substantial economic loss. Viruses appear to be the most common cause of shrimp infections. Significant obstacles in the detection of shrimp infections include symptom similarity in a histopathological examination, multiple viral infections, emerging of new viral disease, and drawbacks of universal molecular techniques such as PCR (supplementary information 1: Figure S1 and S2). Therefore, there is an urge to develop a fast and cheap, *in-situ* diagnosis kit that eliminates the lab-intensive procedures and PCR method. Farmers, exporters and food chain industry can use this way to increase awareness in food safety for people who consume shrimps at large from raw to processed product. In general, this present work describes a diagnostic method that can be used to detect viruses that may be present in an organism, carriers, in an environment, infected samples and other microorganisms while addressing food safety issues, disease surveillance, and epidemiology. On these notes, this current method can demonstrate the use of technology *in vivo* systems, and this will be an application to be used in health and agriculture in general.

The simplicity of the sensing elements are apparent since DNA being a semiconductor-like material, a Schottky diode-like property as can be seen from the I–V profiles. Different types of DNA will, therefore, exhibit distinct I–V profiles due to the base pair sequence variations specific to each type of DNA. Since DNA is a semiconductor-like material, the charge injection property governs the conduction mechanism as discussed by the Schottky diode analysis. The I–V characteristics describing the interface property are therefore the basis for DNA sensing employed in our method.

We believe that this novel electronic profiling technique may prove instrumental towards developing efficient and rapid solid state based sensors for *in-situ* applications in aquaculture and that consist from farm to table as a concept to ensure food safety to people. In general, the specific electronic profiles generated using the DNA-specific Schottky diodes could be utilised to significantly “fingerprint” different DNA sequences and provide a basis for non-laboratory based diagnostics and pathology.

## Methods

### Preparation of viral capsid gene sequence

Four synthetic coding DNA viral genes (Infectious hypodermal and hematopoietic necrosis virus (IHHNV), Monodon baculovirus (MBV), Taura syndrome virus (TSV), Yellowhead virus (YHV) was used for this experiment (supplementary information 3). The synthetic virus was prepared based on the alignment of virus from various geographical isolates. The alignment result showed the conserved region of the virus hence the synthetic virus can be developed based on the best fit sequence data. Therefore, there is no bias towards any isolate from any geographical isolates. Additionally, seasonal outbreak of specific pathogens in this experiment will halt and possibly hamper the experimental progress; therefore, the synthetic viruses which are synthesised based on the real virus sequence can serve as the best candidate in optimising and standardising the condition and result in generating the most accurate I–V curves. Further to this, a calibration curve can be plotted for identifying actual samples from the profile database obtained through this and other similar studies. As such, this preliminary work with the synthetic versions is crucially necessary to be carried out first before electronic profile deviations due to geographical, mutations etc. could be integrated and understood.

The sequencing result of the synthesised capsid virus stated in the report was sequenced for verification, before proceeding with I–V characterisation. The sample is a DNA or cDNA template derived from the RNA of the sample. DNA extraction was carried out using EasyPure® Marine Animal Genomic DNA Kit. Optionally, cDNA is obtained using a TranScript® One-Step gDNA removal and cDNA Synthesis SuperMix manual and TransZol™ Up Plus RNA Kit for RNA extraction.

### Bacterial strain

The *VP*_AHPND_ (NCKU_TV_3HP_AHPND *V. parahaemolyticus*) strain from Thailand used in this study was collected from Department of Fisheries Malaysia. The strain was inoculated and cultured in tryptic soy broth (TSB) (Merck, Darmstadt, Germany) and tryptic soy agar (TSA) (Merck, Darmstadt, Germany) containing 2% NaCl and incubated at 28 °C for 18 h.

### Bacterial DNA extraction

Total genomic DNA was extracted by the modified boiling method^38^. A single colony of the isolate from agar plate was inoculated into tryptone soy broth supplemented with 2% NaCl and incubated at 28 °C for 18 h. One ml of the overnight culture was subjected to centrifugation at 10,000 × g for 5 min to pellet the bacterial cells. The supernatant was discarded before added with 200ul of nuclease-free water. The tube containing bacterial suspension was vortex and boiled for 10 min. The tube was then transferred to ice for 10 min and centrifuged at 10,000 × g for 5 min. The extracted genomic DNA (supernatant) was transferred to a new tube and stored at −20 °C. The concentration of the genomic DNA was measured using a *NanoDrop*system (Software NanoDrop 2000) with spectrophotometric ratios of 1.8 to 2.0 (A_260_/A_280_). To demonstrate the sequence/length dependency of this detection technique, different DNA sequences with the same length were subjected to measurment (supplementary information 4: Figure S4).

### Fabrication of the Al/DNA Schottky barrier diode

ITO slides (width 1.1 mm, the layer thickness of 100 nm with a dimension of 2 cm × 2 cm and 377.0 Ω/sq and about 10^4^ S/cm of sheet resistance and conductivity), purchased from KINTEC, Hong Kong. The Al wire electrodes were used in this work with the thickness of (0.50 ± 0.05) mm and purity of 99.999% (Sigma Aldrich, USA).

**Figure 5:**
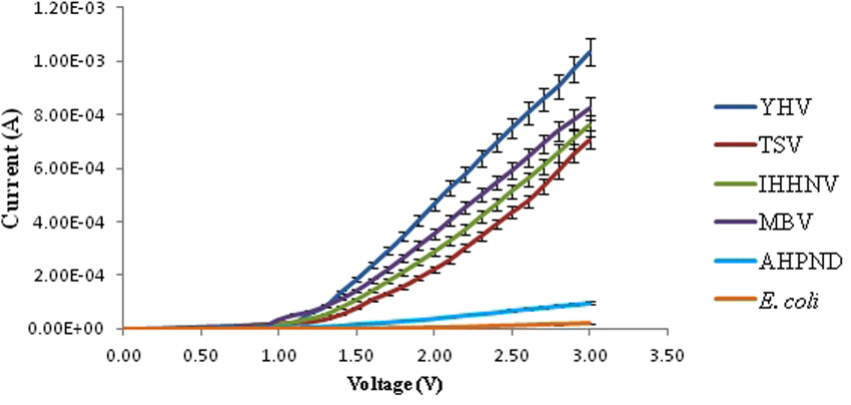
(**a**) Photograph and (**b**) schematic diagram of the DNA-specific Schottky diode fabricated for the study while (**c**) represents the equivalent electrical circuit.

The ITO substrates were thoroughly cleaned by using soap, deionised water, acetone and iso-polypropanol and finally, the slides were dried using nitrogen gas to remove any water and other residues. 10 µl of DNA solution was then applied onto the cleaned ITO substrate using the simple and economical method of self-assembly. The entire preparation and fabrication process was undertaken in a 1 K clean room to make sure the same environmental conditions (temperature at about 21 °C and relative humidity of 70 to 80%) were maintained. Figure 5 demonstrates the schematic diagram and picture of the fabricated sensor for the measurement of the I–V characteristics (Keithley Electrometer, SMU 236).

### Acquisition of I–V characteristic profiles and calculation of electronic parameters

I–V characteristic of the forward bias region was used to obtain electrical parameters such as ideality factor, barrier height and series resistance of Schottky diode. Two different approaches were taken to calculate the values of the electrical parameter, and the values were compared to the most reliable model. The first method is to calculate the electrical parameter with the assumptions that the current in the Schottky diode isbased on thermionic emission, and the effects of series resistance can be ignored for low forward bias region. The equation for voltage and current can be expressed as;

1 $$I={I}_{0}{e}^{\tfrac{qV}{nkT}}[1-{e}^{\tfrac{-qV}{kT}}]$$

for $V > \frac{3kT}{q}$, equation (1) becomes as;

2 $$I={I}_{0}{e}^{\tfrac{qV}{nkT}}$$

where *n* is the ideality factor, *q* is the electronic charge, *k* is the Boltzmann constant, *T* is the temperature in Kelvin and *I*_*o*_ the reverse saturation current which can be expressed as;

3 $${I}_{0}=A{A}^{\ast }{T}^{2}{e}^{\tfrac{-q{\varnothing }_{b}}{kT}}$$

where *A* is the effective diode area, A^*^ is the effective Richardson constant which is equal to 1.3 × 10^5^ A/cm^2^K^2^ for ITO^23,36,39,40^ and *Ø*_*b*_ is the zero-bias barrier height. The *I*_*o*_ can be obtained by extrapolating the straight line of *lnI versus V* to intercept the axis at zero voltage.

4 $$\mathrm{ln}\,I=\,\mathrm{ln}\,{I}_{o}+\frac{q}{nkT}V$$

5 $${\varnothing }_{b}=\frac{kT}{q}\,\mathrm{ln}(\frac{A{A}^{\ast }{T}^{2}}{{I}_{0}})$$

Further, the deviation of Schottky diode form ideal thermionic emission model can be evaluated by calculating the ideality factor, *n* which can be extracted from the gradient of the straight line *ln I* versus *V* plot and can be written from equation (2) as;

6 $$n=\frac{q}{kT}(\frac{\partial V}{\partial (\mathrm{ln}\,I)})$$

To verify the values obtained for ideality factor and barrier height Cheung’s method was adopted which includes the series resistance effect to the calculation. The equations involved in this method can be expressed as;

7 $$\frac{dV}{d\,\mathrm{ln}\,I}=n\frac{kT}{q}+I{R}_{s}$$

Using *dV/dln(I)* versus *I* graph the axis intercept at zero current was obtained to calculate the ideality factor of the Schottky diode. On the other hand, to calculate the value of barrier height, *H(I)* function was used as defined by Cheung which can be expressed as;

8 $$H(I)=V+n\frac{kT}{q}\,\mathrm{ln}(\frac{I}{A{A}^{\ast }{T}^{2}})$$

9 $$H(I)=n{\varnothing }_{b}+I{R}_{s}$$

From the plot of *H(I)* versus *I*, the y-axis intercept was used to estimate the barrier height of the diode. The values were compared with the previous method to verify the effects of series resistance.

## Electronic supplementary material

Supplementary 1-4
