# Publisher Correction: Electronic Properties of Synthetic Shrimp Pathogens-derived DNA Schottky Diodes

**DOI:** 10.1038/s41598-018-24116-5

**Published:** 2018-04-12

**Authors:** Nastaran Rizan, Chan Yen Yew, Maryam Rajabpour Niknam, Jegenathan Krishnasamy, Subha Bhassu, Goh Zee Hong, Sridevi Devadas, Mohamed Shariff Mohd Din, Hairul Anuar Tajuddin, Rofina Yasmin Othman, Siew Moi Phang, Mitsumasa Iwamoto, Vengadesh Periasamy

**Affiliations:** 1 Low Dimensional Materials Research Centre (LDMRC), Department of Physics, Faculty of Science, University of Malaya, 50603 Kuala Lumpur, Malaysia; 2 Institute of Biological Sciences, Faculty of Science, University of Malaya, 50603 Kuala Lumpur, Malaysia; 3 Department of Chemistry, Faculty of Science, University of Malaya, 50603 Kuala Lumpur, Malaysia; 4 Centre for Research in Biotechnology for Agriculture (CEBAR), University of Malaya, 50603 Kuala Lumpur, Malaysia; 5 High Impact Research (HIR) Functional Molecules Laboratory, Institute of Biological Sciences, Faculty of Science, University of Malaya, 50603 Kuala Lumpur, Malaysia; 6 Institute of Ocean and Earth Sciences, University of Malaya, Kuala Lumpur, 50603 Malaysia; 7 Department of Physical Electronics, Tokyo Institute of Technology, 2-12-1 Okayama, Meguro-ku, Tokyo, 152-8552 Japan; 8 Faculty of Veterinary Medicine, Universiti Putra Malaysia, 43400 Serdang, Selangor Malaysia

Correction to: *Scientific Reports* 10.1038/s41598-017-18825-6, published online 17 January 2018

This Article contains an error in the order of the Figures. Figures 1, 2, 3, 4 and 5 were published as Figures 5, 4, 3, 2 and 1 respectively. The correct Figures appear below. The Figure legends were correct from the time of publication.

**Figure 1:**
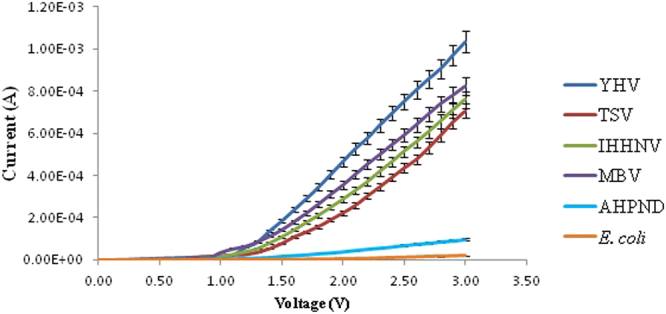
Positive biased I–V profiles for the four types of virus and two bacterial species carried-out in triplicates. A band-like structure is seen for the viruses (top region) and for the bacteria (bottom region).

**Figure 2:**
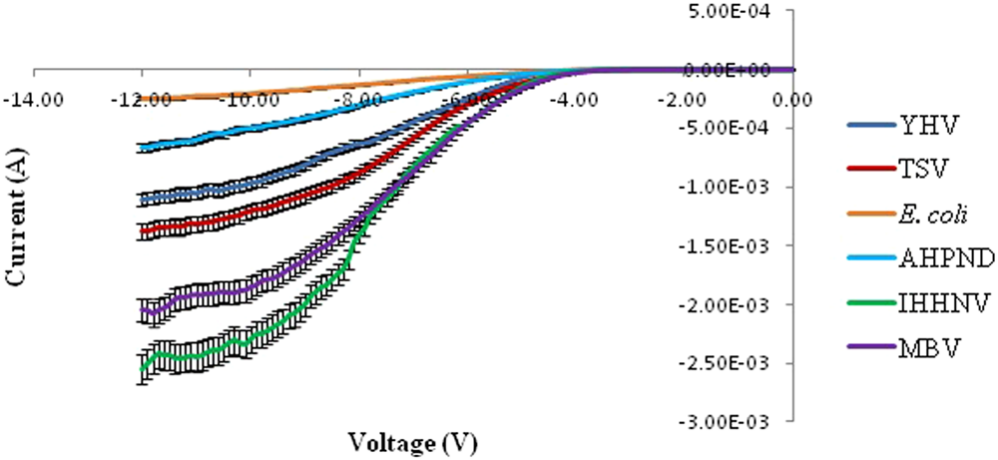
Negative biased I–V profiles for the four types of virus and two bacterial species carried-out in triplicates.

**Figure 3:**
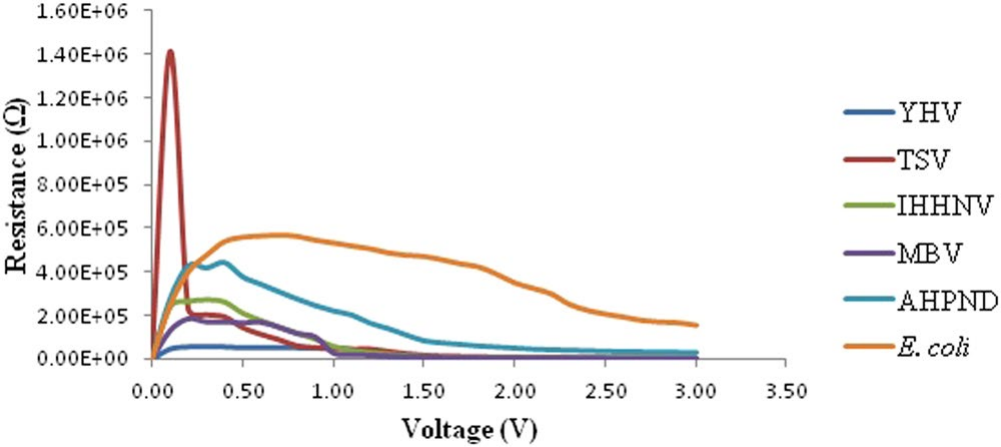
Resistance profile against bias voltage for the four the virus and two bacteria species.

**Figure 4:**
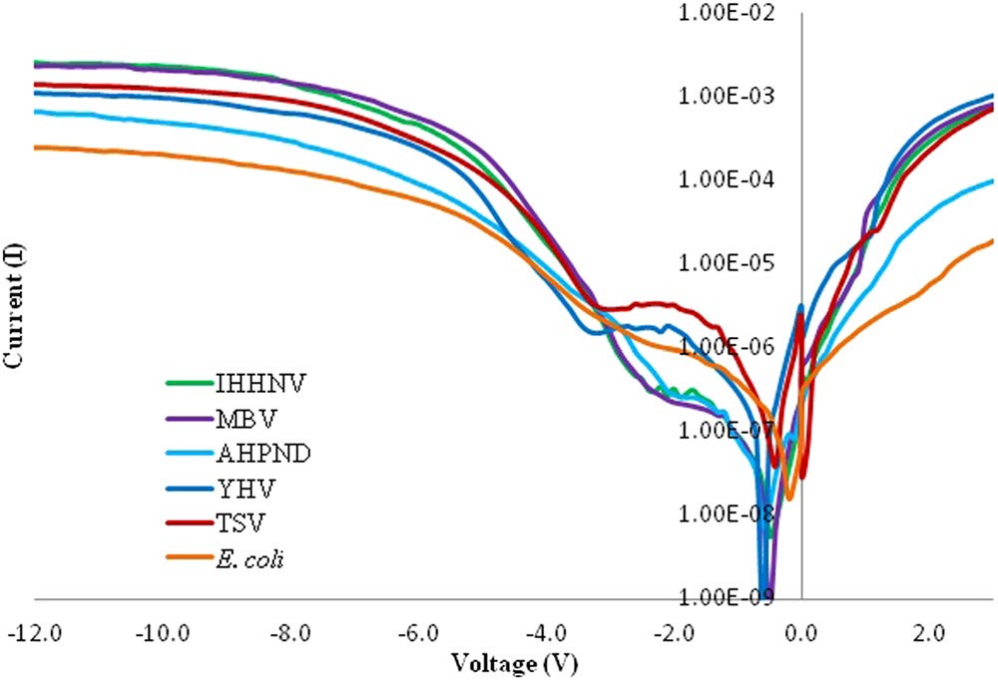
Semi logarithmic I–V characteristics of virus and bacterial species.

**Figure 5:**
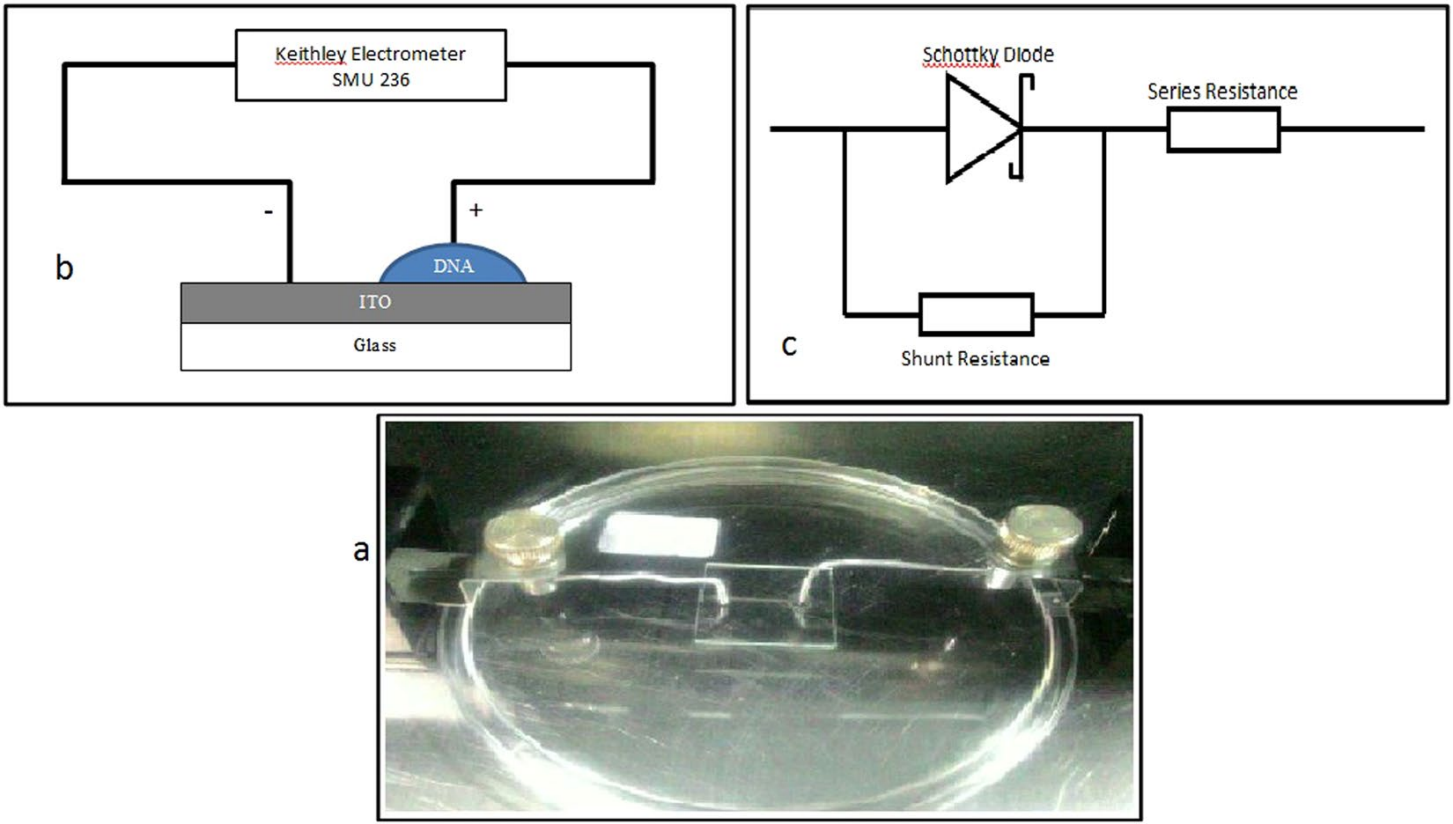
(**a**) Photograph and (**b**) schematic diagram of the DNA-specific Schottky diode fabricated for the study while (**c**) represents the equivalent electrical circuit.

