# Supplementary material for: Electronic Properties of Synthetic Shrimp Pathogens-derived DNA Schottky Diodes
Source: Sci Rep. 2018 Jan 17;8:896. doi: 10.1038/s41598-017-18825-6 (PMC5772554; doi:10.1038/s41598-017-18825-6)
Supplement: Supplementary file 1 — Supplementary 1-4 [file 41598_2017_18825_MOESM1_ESM.pdf]

Electronic Properties of Synthetic Shrimp Pathogens-derived DNA Schottky Diodes

Nastaran Rizan[1,2,3], Chan Yen Yew[1,2,4], Maryam Rajabpour Niknam[1,2,5], Jegenathan Krishnasamy[1], Subha Bhassu[2,4], Goh Zee Hong[2,4], Sridevi Devadas[2,4], Mohamed Shariff Mohd Din[8], Hairul Anuar Tajuddin[3], Rofina Yasmin Othman[2,4], Siew Moi Phang[1,6], Mitsumasa Iwamoto[7] and Vengadesh Periasamy[1*]

[1]Low Dimensional Materials Research Centre (LDMRC), Department of Physics, Faculty of Science, University of Malaya, 50603 Kuala Lumpur, Malaysia

[2]Institute of Biological Sciences, Faculty of Science, University of Malaya, 50603 Kuala Lumpur, Malaysia

[3]Department of Chemistry, Faculty of Science, University of Malaya, 50603 Kuala Lumpur, Malaysia

[4]Centre for Research in Biotechnology for Agriculture (CEBAR), 50603 Kuala Lumpur

[5]HIR functional molecules laboratory, Institute of Biological Sciences, Faculty of Science, University of Malaya, 50603 Kuala Lumpur, Malaysia

[6]Institute of Ocean and Earth Sciences, University of Malaya, Kuala Lumpur 50603, Malaysia

[7]Department of Physical Electronics, Tokyo Institute of Technology, 2-12-1 Okayama, Meguro-ku, Tokyo 152-8552, Japan

[8] Faculty of Veterinary Medicine, Universiti Putra Malaysia, 43400 Serdang, Selangor, Malaysia

***Corresponding Author:** Associate Professor Dr. Vengadesh Periasamy, Low Dimensional Materials Research Centre (LDMRC), Department of Physics, Faculty of Science, University Malaya, Kuala Lumpur, Malaysia. Email: vengadeshp@um.edu.my, Tel: +60-379674038.

Supplementary 1

PCR detection emphasises the designed primers which targets the corresponding sequence. The above results show that the dual priming oligonucleotide (DPO) primers designed targeted on prawn and shrimp viruses. These patented primers were able to detect and differentiate the type of virus infection. A mixture of primers set allows simultaneous detection of 4 types of virus infections through band sizes. While in this paper, we emphasised in using DNA-specific Schottky diodes to generate I-V curve profiles in differentiating the types of virus infection. Different ATCG combination and nucleic acid sequence will generate different I-V curves which can be utilised to differentiate the type of infection. Our results indeed have been compared with the PCR result in generating reasonable data (I/V curve) in concluding the type of I/V curve versus type of infection.

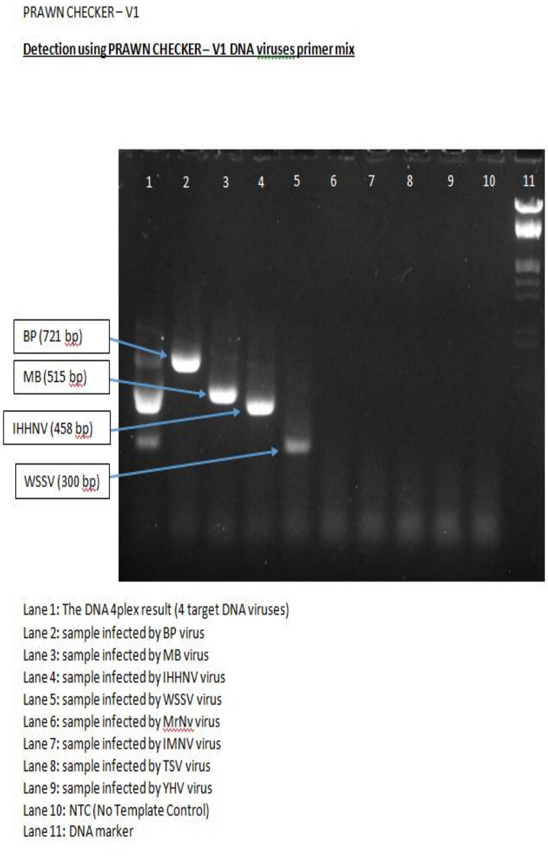

Figure S1. Multiplex of DNA viruses using DPO primers mixture. Lane 1 shows the multiplex bands of 4 multiplex DNA viruses while lane 2 to 5 shows individual bands of DNA viruses with different band size.

Detection using PRAWN CHECKER – V1 RNA viruses primer mix

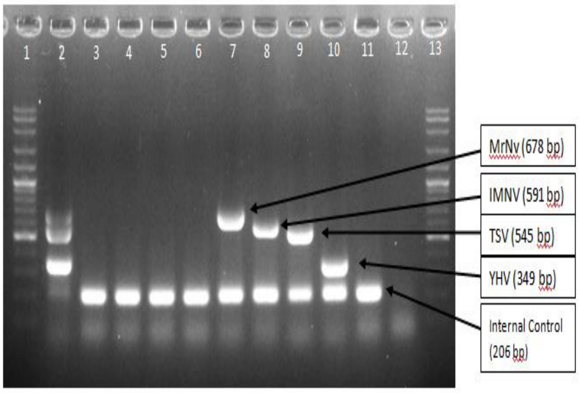

Lane 1 and 13: DNA marker
Lane 2: The RNA 5plex result (4 target RNA viruses with sample cDNA)
Lane 3: cDNA of sample infected by BP virus
Lane 4: cDNA of sample infected by MB virus
Lane 5: cDNA of sample infected by IHHNV virus
Lane 6: cDNA of sample infected by WSSV virus
Lane 7: cDNA of sample infected by MrNv virus
Lane 8: cDNA of sample infected by IMNV virus
Lane 9: cDNA of sample infected by TSV virus
Lane 10: cDNA of sample infected by YHV virus
Lane 11: cDNA of sample healthy sample
Lane 12: NTC (No Template Control)

Figure S2. Multiplex of RNA viruses using dual priming oligonucleotide (DPO) primers mixture. Lane 1 shows the multiplex bands of 4 multiplex RNA viruses while lane 7 to 10 shows individual bands of RNA viruses with different band size.

Supplementary 2

This new technique provides the potential to achieve sensitive and high-throughput species identifications. The simplicity nature of these methods is suitable for the development of a hand-held DNA diagnostic device. The following steps were undertaken to demonstrate the reproducibility and sensitivity of the electronic sensor;

Please refer to the Supplementary Figure below, which explains the positive biased I-V profiles for different concentrations of DNA AHPND (from about 300 ng/ul to 5 ng/ul).

In this technique, we prepared different concentrations of DNA strands to investigate the sensitivity of the sensor operation. Extensive experiments (more than 50 repetitions each) were carried-out to obtain data from different concentrations of DNA (high to lowest levels) to proof the sensitivity of this electronic sensor.

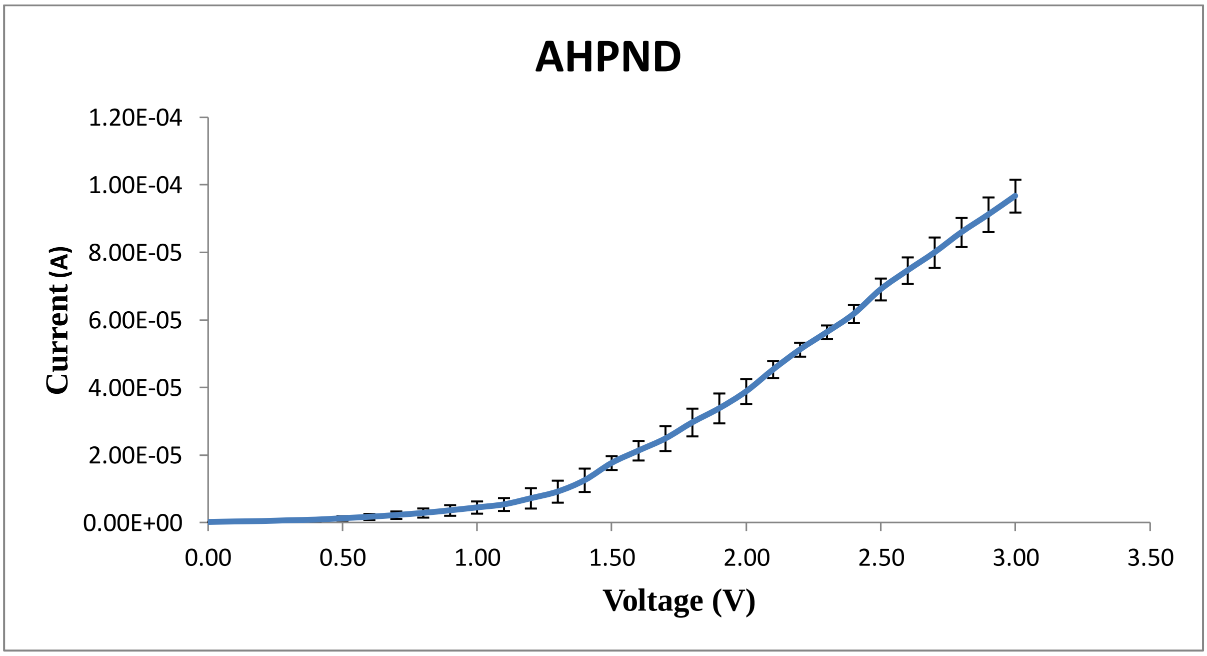

Figure S3: Positive biased I-V profiles for different concentrations of DNA AHPND (from about 300 ng/ul to 5 ng/ul). The small error bars indicate the high repeatability and sensitivity of the experiments carried-out.

Supplementary 3

Please refer to the Supplementary data below, which shows real sequences of our samples

Sequences of viral gene

Infectious hypodermal and hematopoietic necrosis virus (IHHNV) (987bp)

ATGTGCGCCG ATTCAACAAG ATCAAGCCCA AGGAAAAGAT CCAGGAGGGA TGCACATAAT GAAGACGAAG AACACGCCGA GGGATCAAGT GGACCAGACC CACACAGATG TCTACAATTC AATACGGGAG ACTCAATACA TATTACTTTC CAAACAAGAA GATACTTCGA ATTCGACGCT GCCAATGATG GAAACTTCGA CGGAAAAAAC TTATACTGCC TCCCACTACA TTGGATGAAC TTATATCTCT ATGGACTAAA AAGCAGTGAC AGTTCAGCAA CAGAAACACA GCGATATAAG ATGGTAAAGT CAATGATGAA AACCTACGGA TGGAAAGTTC ACAAAGCAGG CGTCGTAATG CACTCAATGG TACCCCTTAT GAAAGACTTA AAGGTATCAG GAGGAACATC ATTTGAAACT CTCACATTTA CAGATACCCC ATATTTAGAA ATATTTAAGG ATACTACTGG ACTACATAAT CAACTAGCAA CTAAGGAAGC CGACGTAACA TTAGCAAAAT GGATACAAAA TCCGCAACTT GTGACAGTAC AATCAACAGC AGCAAATTAT GAAGACCCAA TCCAACAATT TGGATTTATG GAACAAATGC GAACTGGAGA CCGAAAAGCC TATACAATCC ATGGTGACAC TAGAAACTGG TATGGCGGAG AAATACCAAC AACCGGACCC ACGTTCATCC CAAAATGGGG TGGCCAAATG AAGTGGGACA AACCAAGCAT TGGAAACCTA GTCTACCCAG CGGATCACCA TACAAACGAC TGGCAACAAA TCTTCATGAG AATGTCACCA ATCAAAGGAC CAAATGGAGA CGAACTTAAA CTTGGCTGCA GAGTACAAGC CGACTTCTTC CTCAACCTAG AAGTACGACT CCCACCACAA GGATGTGTCT CAAGTTTGGG AATGTTACAA TATCTTCACG TACCATCTAC TGGACAACTT AACAGATGTT ATATTATGCA TACTAAC

Monodon baculovirus (MBV) (600bp)

ATGTTCGACG ATAGCATGAT GATGGAAAAT ATGGACGACC TTAGTGGAGA TCAGAAGATG GTGCTCACAC TTGCTGCGGC TGGTGCTGTG GCTGGAGCAT CGAAGATGTT GAACGAAGCT GCAGACCTGA AGAAAAATTA CAAGGATACT CCACTTGAAG AATATTTCAA AGATAAGTAT TCAGGCAACA AAAAAAGAAA GATCACTGAT CAGGAATTTG AACTCCCTAA GTCTATTGAT CCACTTGAAA ATCATTTCAA AGGACTGTCC CGTCCTCGTG TAGGCCCTCG AATGGCAAAA CAGCTTGCAA ATAAAATGAG TGACAACAAA ATGCATTATA AATTTAACAG CTTTCAGACA AATAAACACT TTAATACTCA CACAATTTAC AAGCGAACAA ATCTCACTTC TTCTAAACTA ATGGGCTTTT CGGGTCAGAG TGATTTTGGC GTACCCAAAT ACAACAGTGC AGTCACACTT CCTCTGGAAG TATTGGAATT TTGGGTAGGT GACAACACAA ATCCTAATGT TGAACATTCT AAGGGTAGTA TGGCATTGAA AAATAGTGAA TGTATGATTG CATCTATGAA ACTTAAACTT

Taura syndrome virus (TSV) (900bp)

AACATCAAAC TATATGGGCA TGCCCCATCT GTGACATCTT CAGTATATCC GTCTACTCAG TCCGGATATG ATGATGATTG TCCCATTGTG CATGCGGGAA CTGATGAGGA TTCTTCTAAA CAGGGGATTG TCTCAAGGGT TGCAGACACC GTTGGTGCGG TGGCAAATGT AGTAGATGGG GTAGGAGTAC CTATTCTATC CACAATTGCC AAGCCTGTTT CCTGGGTGTC GGGCGTAGTG AGTAATGTAG CTTCAATGTT CGGATTTTCA AAAGATAGGG ATATGACGAA AGTCAACGCA TATGAGAACT TACCTGGTAA GGGCTTCACT CATGGTGTTG GCTTCGATTA TGGCGTACCC CTGTCTCTTT TCCCTAACAA TGCCATTGAT CCCACAATTG CAGTGTCTGA AGGATTAGAT GAAATGTCTA TTGAATACCT AGCACAGCGA CCATATATGC TCAACAGATA CACTATCAGA GGTGGTGACA CTCCTGATGA ACATGGAACA ATTATTGCAG ATATTCCAGT GAGTCCTGTC AATTTTAGTT TGTATGGTAA AGTTATTGCT AAGTATCGCA CCCTATTCGC TGCCCCAGTT AGTCTAGCTG TAGCAATGGC TAATTGGTGG CGTGGAAATA TTAACCTTAA TCTTCGCTTT GCTAAGACGC AGTACCATCA ATGCAGATTG CTGGTGCAAT ATCTCCCCTA TGGTAGTGGT GTTCAACCAA TAGAAAGTAT CCTTTCACAG ATCATCGACA TCTCACAAGT CGATGATAAG GGTATTGACA TTGCTTTTCC TTCCGTCTAT CCCAATAAGT GGATGCGAGT GTACGATCCA GCGAAAGTTG GGTACACGGC AGATTGTGCC CCAGGCCGAA TCGTCATTTC CGTTCTCAAC

Yellowhead virus(YHV) (684bp)

ATGAACCGTC GTACACGCAC CGCAACTCCT ATGCCTCGTC GTCGCCTACC TCCTTCCAAC CGACTCACTC GCAATGCAAG GCTCATCGAG ATTCCTCAAT CCTTCGCAGT CGAACGCGGA AATGGATGGA TGTTGGCATA TGCCCCAGGT AAAAATCCAC TACCGGGAAA AGTCATCGCT CGTATGCAGG CATCTCCATT CATTCAAGGA CTTCAAGAAC AATCCCTCCA AGTTGTCAAG TCATCTGATG GTAAGTATTC AATTTCAAAG AGATACGGTA AAATGGCCAT CACCTATCTT AATCCCAACG ATCCCATTCT GCCAAAGCGT TCAACACAGA AGTCAATCGT TCCCGATCCT TCCCTTGACA TAGAGAACCT AGCTGAAGGT ATCCACGCAA TGAGCCTTGA AGACGACGAA CCCATGGAAA CACAATCA

Supplementary 4

The electronic method employed in this work is highly accurate and will strongly depend on the electronic pathway presented to regarding the resistance pathway provided by the base sequence. Due to the nature of current conduction mechanism, it is well understood that conductivity signature will only be influenced by the "least resistance" pathway which will strongly be dependent on both the base sequence and number of base pair. In this context, it is understood that with different base sequences but with a same number of base pairs, each of the 2 DNA sequences demonstrate characteristic or fingerprinting electronic profiles unique to their charge pathway mechanism.

However, we still carried-out the necessary experiment to demonstrate the variations that arise even within different DNA sequences but with the same number of base pairs shown below; please refer to the supplementary Figure below:

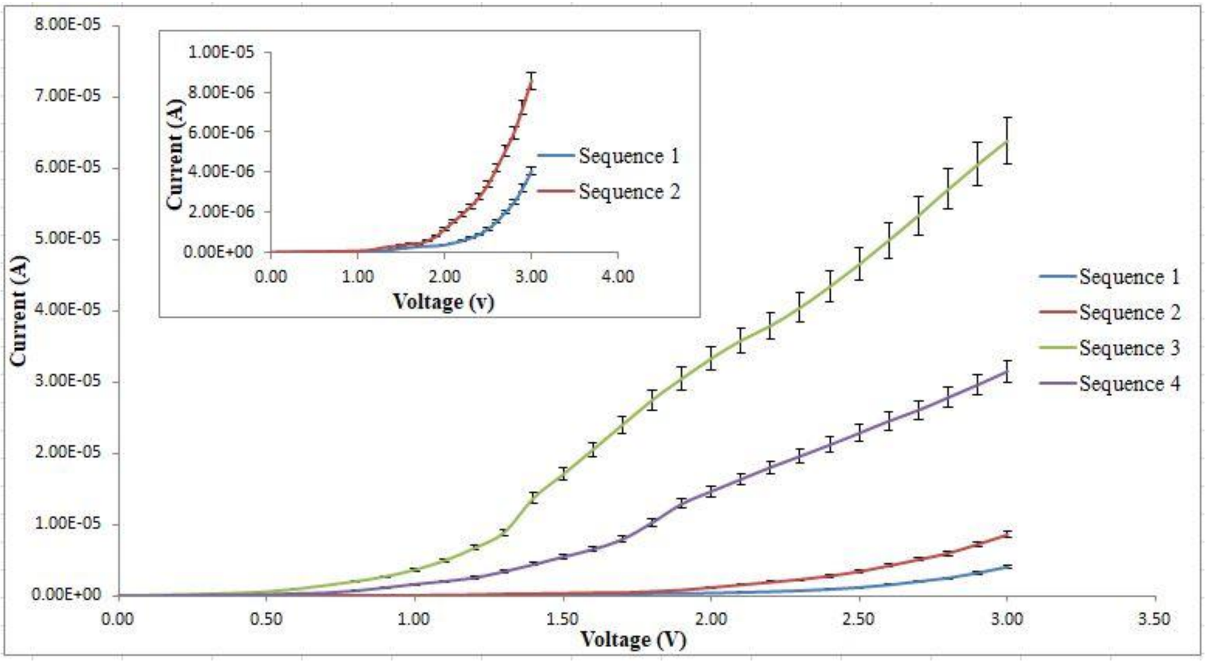

S4: Four sets of complementary strands of DNA with a length of 20bp were designed, synthesized and subjected IV characterization. Each set had a different sequence. Sequence 1 is rich in GC bases (GC=70%), sequence 2 is rich in AT bases (GC= 25%) while sequence 3 and sequence 4 have same GC bases (GC=50%). These sequences exhibited different electronic profile.

Sequence 1

5'   GCA TTC GGC GCG GCG TAA CG   3'

3'   CGT AAG CCG CGC CGC ATT GC   5'

Sequence 2

5’	ATA CCT GTC AGA AAT TAA TA	3’
3’	TAT GGA CAG TCT TTA ATT AT	5’

Sequence 3

5’	GCA TGA TCC CTG ACT ATG TC	3’
3’	CGT ACT AGG GAC TGA TAC AT	5’

Sequence 4

5’	TAA GCA CGT CTG ACC ATG TC	3’
3’	ATT CGT GCA GAC TGG TAC AG	5’
